# PRESIDENTIAL SYMPOSIUM: MUSEUMS AND AGING: NOVEL NETWORK OPPORTUNITIES TO SUPPORT OPTIMAL AGING

**DOI:** 10.1093/geroni/igz038.1318

**Published:** 2019-11-08

**Authors:** Desmond O'Neill, Robert Roush, Robert Roush

**Affiliations:** 1 Trinity College Dublin, Dublin, Ireland; 2 Huffington Center on Aging, Baylor College of Medicine, Houston, Texas, United States

## Abstract

Museums represent an evolving and under-recognized network of opportunity for examining aging while supporting optimal aging across the lifespan. Museums bind communities together in a civic body by “…identifying its highest values, its proudest memories, and its truest truths.”(Duncan, 1991). They represent a secular ritual of the modern state in which the spiritual heritage of the nation is offered as a public reinforcement of political values. Art museums are also sites which enable individuals to achieve liminal experience - to move beyond the psychic constraints of mundane existence, step out of time, and attain new, larger perspectives. The interaction and potential synergies between museums and aging have been insufficiently explored in gerontological scholarship, with the existing emphasis largely focussing on facilitating access to older people and those with age-related health conditions. This symposium reflects and magnifies the networking of GSA with a major art museum through an Educational Site Visit during GSA 2019 to the Blanton Art Museum. It proposes to review museums and ageing in a broader context, exploring the context within which aging is represented in the discourse of heritage and museums, museums networking to provide a repository of late-life creativity, networks of older people as a key resource and client group for museums, life-course and inter-generational engagement with museums. Finally, the insights that the ageing of art works provide for curating the longevity dividend through developing scholarly networks between gerontologists and curators.

